# How Religion, Social Class, and Race Intersect in the Shaping of Young Women’s Understandings of Sex, Reproduction, and Contraception

**DOI:** 10.3390/rel12010005

**Published:** 2020-12-23

**Authors:** Laura M. Krull, Lisa D. Pearce, Elyse A. Jennings

**Affiliations:** 1 Department of Sociology, St. Norbert College, De Pere, WI 54115, USA; 2 Department of Sociology, University of North Carolina—Chapel Hill, Chapel Hill, NC 27599, USA; 3 Center for Population and Development Studies, Harvard T.H. Chan School of Public Health, Boston, MA 02115, USA

**Keywords:** religion, race, social class, contraceptive knowledge, reproductive knowledge, complex religion

## Abstract

Using a complex religion framework, this study examines how and why three dimensions of religiosity—biblical literalism, personal religiosity, and religious service attendance—are related to young women’s reproductive and contraceptive knowledge differently by social class and race. We triangulate the analysis of survey data from the Relationship Dynamics and Social Life study (RDSL) and semi-structured interview data from the National Study of Youth and Religion (NSYR) to identify and explain patterns. From the quantitative data, we find that all three dimensions of religiosity link to young women’s understandings of sex, reproduction, and contraception in unique ways according to parental education and racial identity. There is a lack of knowledge about female reproductive biology for young women of higher SES with conservative Christian beliefs (regardless of race), but personal religiosity and religious service attendance are related to increased contraceptive knowledge for young black women and decreased knowledge for young white women. From the qualitative data, we find that class and race differences in the meaning of religion and how it informs sexual behavior help explain results from the quantitative data. Our results demonstrate the importance of taking a complex religion approach to studying religion and sex-related outcomes.

## 1. Introduction

Even as teenage pregnancies decline, sexually transmitted infections (STIs) have been on the rise across age groups, prompting scholars to continue investigating social and cultural factors shaping adolescents’ contraceptive and reproductive health knowledge (CDC 2019; Martin, Hamilton, Osterman, Driscoll, and Drake 2018). A great deal of research suggests that religious involvement during adolescence lowers the risks of early pregnancy and STIs (Burdette and Hill 2009; Jones, Darroch, and Singh 2005); however, some elements of religion, like conservative religious beliefs or affiliation, have been shown to elevate these risks (Coleman and Testa 2008; Harrington et al. 2014). When attention is paid to what might be the mechanisms for either the protective or the deleterious effects of religion, the focus is primarily on attitudes toward sex, although research suggests that knowledge about sex, reproduction, and contraception is important as well. This knowledge contributes to the formation of efficacious behavioral strategies. Thus, it is important to explore whether specific religious beliefs or practices might be related to the accuracy of one’s reproductive and contraceptive knowledge.

Another largely unexplored aspect of the link between religion and sexual behavior is how social class and race modify the impact of religiosity on sexual behavior and knowledge (Pearce, Uecker, and Denton 2019). In the U.S., due to a long history of White people and their religious institutions, especially those with more financial resources, excluding people of color and the poor or less educated from participation and leadership, religious institutions are highly segregated by both race and social class today (Edwards 2008). Styles of worship and social issues of concern vary across different traditions and congregations (Nelson 2008). Therefore, messages, role-modeling, stigma, and sanctions regarding sex, pregnancy, and contraception are likely to differ across religious traditions and congregations. In addition, social inequalities, including racism in healthcare contributing to heightened levels of medical mistrust, have contributed to unequal access to sexual and reproductive knowledge and resources by social class and race (Gory 2019; Jaiswall and Halkitis 2019; Kusunoki et al. 2016; Rocca and Harper 2012; Weitzman et al. 2017). Thus, it is likely that associations between religious beliefs or practices and reproductive or contraceptive knowledge are moderated by social class and race.

In this study, we use a complex religion framework to examine how young women’s religious beliefs and public and private religiosity relate to their reproductive and contraceptive knowledge. By examining the intersection of religion, race, and social class, rather than simply controlling for the latter two, we can more precisely specify the unique impact of religion on reproductive and contraceptive knowledge (Wilde and Glassman 2016; Wilde and Tevington 2017; Wilde 2017). To analyze how religious influence might be moderated by social class and race, we examine religion’s role separately by levels of parental education and by comparing White and Black women. Further, we examine religion’s role across different combinations of parent education and race. We first present estimates of these relationships quantitatively, using survey data from the Relationship Dynamics and Social Life (RDSL) study. We then present results from a concurrent examination of qualitative data from in-person, semi-structured interviews with a similarly aged group of young women who participated in the National Study of Youth and Religion (NSYR). In our discussion of the findings, we integrate the two sets of results for a fuller understanding of social class and racial complexities in how religious beliefs and practices are related to young women’s understandings of sex, reproduction, and contraception.

## 2. Religion and Knowledge about Reproduction and Contraception

By age 20, 79 percent of all young women in the U.S. have had sexual intercourse, and 78 percent of the women who have had sex used a method of contraception the first time (Martinez and Abma 2015). Most youth – religious or not – hold positive attitudes toward contraception, and teenage contraceptive use has increased over time, although use remains inconsistent (Lindberg, Santelli, and Desai 2016; Ryan, Franzetta, and Manlove 2007). Few young adults oppose birth control on moral grounds, and many believe that people should use protection when having sex (Regnerus 2007). Religiosity is consistently related to important differences in sexual and contraceptive attitudes and behaviors, but we know less about how different aspects of religion shape reproductive or contraception knowledge, a unique and influential psychosocial factor shaping risk for unintended pregnancy and STIs.

Not all dimensions of religion appear to impact sex-related outcomes in the same way or to the same extent, suggesting that the operationalization of religiosity matters for understanding links between religion and reproductive and contraceptive knowledge (Burdette 2009; Burdette, Hill, and Myers 2015; Regnerus 2007; Rostosky, Regnerus, and Wright 2003). Below we focus on three commonly examined dimensions of religiosity--religious ideology, personal religiosity, and religious service attendance—and how each might be related to young women’s understandings of sexual reproduction and contraception.

### 2.1 Religious Ideology

Research on religion and sexuality often focuses on beliefs associated with conservative Christianity, which tends to teach normative and moral ideas about sex, rather than information about safe sex or sexual health, and to emphasize the Bible as a source of moral authority (Burdette, Hill, and Myers 2015; Regnerus 2005, 2007). Conservative Protestants generally encourage sexual abstinence until marriage, heterosexuality, and procreation, and implicitly discourage sexual activity in other contexts (Burdette, Ellison, and Hill 2005; Pearce and Thornton 2007). Research consistently shows that holding conservative religious beliefs regarding family and sex predict a delay in becoming sexually active for adolescents, but that delay rarely lasts until marriage (Adamcyzk 2012; Jones, Darroch, and Singh 2005).

Furthermore, upon becoming sexually active, religiously conservative adolescents are often less knowledgeable about reproductive and sexual health, putting them at greater risk of having unprotected sex (Harrington et al. 2014). Thus, although these adolescents nevertheless tend to support contraceptive use in the abstract, they are still at heightened risk of unprotected sex due to lack of knowledge, lack of preparation (i.e. because they did not plan to have sex, they may not be on birth control or have access to contraceptives), or ambivalence toward pregnancy (Coleman and Testa 2008; Frost, Singh, and Finer 2007; Kusunoki and Upchurch 2011; Manlove, Ryan, and Franzetta 2007; Shattuck 2019). Finally, within Christian traditions there is important variation in contraceptive use, suggesting that holding conservative beliefs does matter: Mainline Protestant youth report the highest level of consistent contraceptive use, with 78% saying they use protection every time, compared to 62% of evangelical Protestants and 57% of Black Protestants (Regnerus 2007; see also Kramer, Hogue, and Gaydos 2007).

One specific religious ideology that has been examined in relation to sexual behavior is whether one’s religion serves as a primary source of moral authority. Regnerus (2010) found that the 11% of adolescents who say they turn first to God or their religious scriptures for moral guidance report having sexual intercourse or oral sex, while 30% of adolescents who say they make decisions based on their happiness are sexually active. Among adolescents who are sexually active, those who say they turn first to God or to scripture report the lowest rates of consistent contraceptive use and are most likely to report never using contraception, compared to adolescents who say they make decisions based on what they have learned from authority figures or what will make them happy (Regnerus 2007, 2010).

A related aspect of religious ideology that has yet to be examined in relation to reproductive or contraceptive knowledge is biblical literalism. A person who is a biblical literalist is someone who believes the Bible to be the inerrant word of God and, as a result, regards the Bible as a clear source of authority for determining what is moral and what is immoral (Hoffman and Bartkowski 2008; Ogland and Bartkowski 2014); it is distinct from a measure of one’s source of moral authority because it is operationalized as a continuum between believing the Bible to be the literal word of God and believing it to be a book of fables (Franzen and Griebel 2013). Religious traditions with a higher percentage of people reporting biblical literalist beliefs, such as evangelical Protestants, are more likely to be anti-premarital sex, anti-abortion, and to view procreation as the primary goal of sex (Burdette, Ellison, and Hill 2005; Pearce and Thornton 2007). Thus, biblical literalism may also be correlated with reproductive and contraceptive knowledge, with people holding a literalist view being less knowledgeable, given how Biblical literalism shapes ideas about pregnancy and family formation.

### 2.2 Personal Religiosity

Personal religiosity, a combination of private practices, like prayer, and religious salience – or how important religion is in an individual’s daily life – is often the most significant predictor of adolescent sexual activity and sexual health knowledge (Burdette and Hill 2009; Burdette, Hill, and Myers 2015; Regnerus 2007; Rostosky, Regnerus, and Wright 2003). This may be because, unlike with attendance and beliefs, which are often shaped by parental involvement and expectations, adolescents may have more control over how central religion is to their daily lives. Adolescents who report high levels of personal religiosity generally have more conservative attitudes toward sex and less objective knowledge about reproductive health and contraception (Coleman and Testa 2008; Crosby and Yarber 2001; Lefkowitz et al. 2004). Higher personal religiosity is associated with delays in first sex and in non-intercourse sexual touching (Burdette and Hill 2009; Jones, Darroch, and Singh 2005).

Personal religiosity may also tap into adolescent perceptions of how closely they follow their religion’s teachings. Gold et al. (2010) find that adolescents who report that their religious beliefs impact their sexual behavior and contraceptive use were more likely to have conservative attitudes toward sex and to question the efficacy of condoms. However, this does not necessarily mean that adolescents take their religious beliefs into consideration when making decisions connected to sexual activity; when asked directly about how much religious beliefs play a role in such decisions, 47% and 56% report their beliefs do not affect decisions about sex or about preventing pregnancy, respectively (Gold et al. 2004). Therefore, reporting a high level of personal religiosity may not necessarily translate to lower levels of reproductive or contraceptive knowledge if, for example, adolescents do not necessarily see their religious beliefs and sexual practices as linked.

### 2.3 Religious Service Attendance

Religious attendance – both parental and/or adolescent attendance at worship services – is consistently found to predict primarily one sexual behavior: timing of sexual debut. Studies show that when parents and/or adolescents attend religious services more frequently, adolescents are more likely to wait longer to have both first sexual intercourse and first non-intercourse sexual encounter (Burdette and Hill 2009; Jones, Darroch, and Singh 2005). However, there appears to be very little direct effect on other behaviors relative to sexual activity, such as contraceptive use or number of partners (Jones, Darroch, and Singh 2005; Kramer et al. 2007). Frequently attending religious services – particularly ones that talk about marriage, abstinence, or conservative sexual attitudes in general – may indirectly negatively impact contraceptive and reproductive health knowledge, given that adolescents may be implicitly encouraged to associate sexual activity with procreation and discouraged from learning more about regulating fertility (Borch, West, and Gauchat 2011; Gonsoulin 2010; Yancey and Emerson 2018).

## 3. Factoring in Complex Religion

Although repeatedly used and supported in prior studies, the general theories outlined above about how three dimensions of religion might be associated with young women’s reproductive and contraceptive knowledge make little consideration of how these linkages might vary by social class or race. In fact, religious ideology and levels of personal and public religious practice and importance vary by social class and race, as do sexual attitudes, knowledge, and behaviors (Baunach 2012; Rocca and Harper 2012; Schnabel 2020; Schwadel 2011).

To enable us to examine more precisely the intersections of religion, race, and social class, we draw on an emergent framework in the sociology of religion – complex religion (Wilde and Tevington 2017; Wilde 2017). This framework urges the analysis of how race, class, and religion combine to influence outcomes rather than simply controlling for race and class in studies of religious influence (Wilde and Glassman 2016; Wilde and Tevington 2017; Wilde 2017). For example, focusing only on differences in socioeconomic status between religious traditions would overlook ways that Blacks and Whites in the same religious tradition nevertheless hold different socioeconomic positions (Wilde and Tevington 2017). Similarly, in the present study, looking only at how understandings of sex, reproduction, and contraception vary by religiosity would obscure how religion may shape Black adolescents’ knowledge differently than White adolescents’ knowledge. Using a complex religion framework allows a clearer picture of inequality, as we can intentionally look not just between groups (i.e. how Black Christian adolescents compare to White Christian adolescents) but also within groups (i.e. how do highly religious Black Christian adolescents compare to nominally religious Black Christian adolescents) (Schnabel 2020).

### 3.1 Considerations of Social Class

Religious affiliation and levels of some practices and beliefs vary by social class (Schwadel 2020; Wilde, Tevington, and Shen 2018). Furthermore, some aspects of social class do not relate uniformly to religiosity. For example, as education increases, the likelihood of believing the Bible to be literal decreases (Schwadel 2011); of those who believe the Bible to be the literal word of God, 56% have a high school degree or less, compared to only 11% who have a college degree (Pew Research Center 2014). However, as education increases, both religious salience and attendance are higher (Schwadel 2011).

Given the way religious characteristics vary by social class, it is likely that the effects of belonging to a more conservative religious group or having conservative beliefs varies by social class. When it comes to attitudes toward poverty or racial inequality and their remedies, scholars have found higher SES Protestants to be less supportive of structural solutions, yet, lower SES Protestants are more supportive of economic restructuring policies (Edgell and Tranby 2007; Hadden 1969). For example, Clydesdale (1999) finds that although there is a positive relationship between biblical literalism and support for systemic solutions to poverty, biblical literalists with a college degree or higher hold a more status-reinforcing perspective—that individuals should solve their own economic problems rather than rely on help from the state. Thus, we may find that among young women who are biblical literalists, those with higher SES backgrounds may be particularly invested in the personal responsibility a person holds for their own sexual morality.

In the general theorizing about religion and sex-related outcomes, scholars argue that more conservative religious traditions will portray premarital sex as wrong and thus downplay (even ridicule) the importance of youth having knowledge about reproduction or contraception (Miller and Gur 2002; Regnerus 2005). We should not, however, expect this emphasis on an ‘abstinence only and thus no need for information’ approach to exist unilaterally across levels of social class.

Scholars have found that growing up in poverty predicts a stronger desire for pregnancy, which might lead to a diminished perceived need for reproductive or contraceptive knowledge (Higgins and Browne 2008; Weitzman et al. 2017). Living in a social environment where early childbearing is destigmatized and supported by the community can indirectly encourage women to be less consistent in their contraceptive use, as the consequences of young childbearing are less pronounced. When women live in areas with high levels of poverty and few educational and career opportunities, teenage childbearing may have a minimal effect on their future prospects, resulting in lower motivation to use contraception consistently; in contrast, teenagers in well-resourced neighborhoods may face stigmatization from peers and may have reduced educational and occupational outcomes as a result of teenage pregnancy, suggesting they may have more to lose (Campa and Eckenrode 2006; Diaz and Fiel 2016; Edin and Kefalas 2005; Gorry 2019; Weitzman et al. 2017). This fear of stigmatization would be especially heightened for those who are a part of higher SES conservative religious communities or who have strong conservative beliefs themselves, for it would be a sign of failing to observe the perceived call from God to be abstinent before marriage, in addition to putting their futures in jeopardy.

Religious involvement and personal religiosity are also experienced in different ways by social class, which can then translate to different mechanisms of religious influence. In recent work, Lee and Pearce (2019) demonstrate unique relationships between religious involvement and educational outcomes by social class. Youth who have parents with higher education and income tend to view religion as more of a family affair, a social activity, and one of a variety of social contexts in which they are safe and encouraged by adults to aspire to a college degree and beyond. For the most part, higher SES youth do not feel that there is anything particularly special about the role religious involvement plays in their lives. On the other hand, youth from less socioeconomic advantaged families emphasize the key role of their religious faith as a motivator to keep striving for academic success and a coping mechanism for when challenges arise.

Similarly, religious involvement or personal religiosity might not be as influential over the reproductive or contraceptive knowledge of higher SES young women as for lower SES women. Higher SES women will have greater access to higher quality information in general, and holding the nature of one’s belief in biblical literalism constant, religious service attendance and personal religiosity, such as prayer or the importance of religion, are unlikely to encourage the seeking of knowledge any more or less. Then, for young women with more socioeconomically disadvantaged backgrounds, although religious involvement and personal religiosity might encourage seeking knowledge to help protect against unintended pregnancy or sexually transmitted infections, the adults in their congregations may not have as accurate information to share. Indeed, most working-class women report using no contraception at first sex, whereas nearly all middle class women do (Higgins and Browne 2008). Women at lower socioeconomic levels face more financial barriers and time disparities in acquiring contraception and possibly the knowledge that goes along with it (Higgins and Browne 2008).

### 3.2 Considering Race

In addition to social class variance in the relationship between religion and understandings of sex, reproduction, and contraception, we must also consider race. At the intersection of race, religion, and sexuality, research consistently shows that Black adolescents are both more religious and more sexually active than their white peers (Blum et al. 2000; Regnerus 2010; Rostosky et al. 2004). Compared to their white peers, Black adolescents are more likely to report a high level of attendance and personal devotion during adolescence, with any religious decline more likely to occur in young adulthood (Lee, Pearce, and Schorpp 2017). Black adolescents are also most likely to be affiliated with historically Black or evangelical Protestant congregations, and accordingly they may report high levels of Biblical literalism: the Pew Research Center found that 60% of people affiliated with historically Black Protestant churches believe the Bible to be the literal word of God, followed closely by evangelical Protestants at 55%, while only 24% of Mainline Protestants report being literalist (Pew Research Center 2009). Furthermore, religious teachings around gender norms that encourage male headship may leave some Black women feeling unprepared or unable to navigate contraceptive use in their relationships; in response, some Black women are challenging their churches to destigmatize pregnancy outside of marriage and to empower women in their sexual decision making (Piper et al. 2020).

Black families attend religious services more frequently than others (Manlove et al. 2006); in 2014, 47% of Black adults reported attending at least once a week, and another 36% reported attending about monthly; in contrast, only 34% of white adults report weekly attendance, with 32% attending about monthly (Pew Research Center 2014). Black women report higher levels of religious salience than white women, with important variation by social class and sexuality (Schnabel 2018, 2020).

Importantly, several studies show that religion appears to affect Black and White women differently. Manlove et al. (2006) find that more frequent parental attendance delays first sex for all racial and ethnic groups except for Black adolescents, and Regnerus (2010) finds that highly religious Black adolescents reported higher levels of sexual activity than their less religious Black peers and their highly religious white peers, while highly religious whites reported lower levels of sexual activity than their less religious counterparts. However, for sexually active Black teenagers, religion may be a protective factor, as some young women reported better communication with partners and safer sex practices (McCree et al. 2003). Given this, a potential combination of religious stigma against teenage pregnancy and access to informal support and education within congregations may result in young religious Black women taking more steps to gain the knowledge necessary to avoid pregnancy and STIs.

Finally, when analyzing race and reproductive health and knowledge, we must consider how the long history of racism, prejudice, and discrimination in medicine produces health inequalities and contributes to high levels of medical mistrust among people of color (see Jaiswall and Halkitis 2019 or Prather et al. 2019 for recent comprehensive reviews on racism in medicine and medical mistrust). Furthermore, Black women seeking medical advice about reproductive health may experience particularly high levels of medical mistrust, due to a history of nonconsensual experimentation and forced sterilization (Prather et al. 2018); studies also show that high levels of medical mistrust contribute to Black women, more than White women, reporting that they feel uncomfortable discussing sexual health-related concerns with doctors (Rosenthal and Lobel 2018; Tekeste et al. 2018).

This history of racism and discrimination undoubtedly impacts any racial differences in contraceptive knowledge and use that exist between Black and non-Black women. Some studies show that Black and white women generally have similar levels of contraceptive knowledge, but they vary in their beliefs about contraception (Rocca and Harper 2012; Kusunoki et al. 2016). For example, Black women are more likely than white women to believe that hormonal contraceptives, such as the pill, negatively impact sex drive or pose health risks, although these differences largely disappear after controlling for social class and having health insurance (Guzzo and Hayford 2012). There is less agreement around potential differences in use; some studies find that Black women use contraceptives less consistently than white women (Rocca and Harper 2012), while others find that there is no difference in use, but rather differences in what methods are used (Kusunoki et al. 2016; Shih et al. 2011). Young whites prefer the pill, which is generally more effective at preventing pregnancy than the male condom, which young Blacks prefer (Kusunoki et al. 2016). Thus, Black women may know more about condom use than White women. Given all this, we must recognize that racial differences in religiosity and in sexual practices may mean that religion differentially impacts Black women’s contraceptive and reproductive health knowledge relative to White women’s knowledge.

### 3.3 Considering Race and Social Class Simultaneously

The complex religion approach is motivated by complex inequality, or the idea that structures of inequality overlap, and the overlaps that characterize people’s lives will shape their outcomes (Wilde and Glassman 2016; Choo and Ferree 2010). Thus, in our analysis, instead of just focusing on religion’s intersection with one aspect of inequality, we examine two. We ask how religion, social class, and race interact to shape young women’s understandings of knowledge about sex, reproduction, and contraception. We could find no existing research on how race and social class might interact to further complicate the relationship between religion and sexual attitudes, knowledge, or behavior. However, it is useful to apply what is known from other studies of the intersection of race and social class, when it comes to religion. One example is the work of Edgell and Tranby on racial attitudes. They find that the highest support for understanding racial inequality as a systemic problem comes from religiously active Black women with lower education, while the lowest support for a structural perspective comes from more highly education, conservative Protestant women who attend frequently. Thus, we also expect that comparisons that simultaneously consider religion, social class, and race will reveal differences in young women’s reproductive and contraceptive awareness.

We expect that conservative beliefs like biblical literalism might result in low levels of reproductive or contraceptive knowledge among women from higher SES families, and that this might be especially pronounced among white women. This is because issues of personal sexual morality (e.g., the virginity pledge movement) have been more predominant in white religious communities than in Black religious communities. When premarital abstinence is emphasized, parents sometimes block access to information about sex and contraception (Kumar and Brown 2016; Wilkinson et al. 2018). In addition, Black religious communities have a history of educating on public health issues such as HIV and promoting means of prevention. And, socioeconomically disadvantage youth have been shown to rely on religion to help them build human capital and avoid life events, such as unintended pregnancy, that might slow them down (Lee and Pearce 2019). Thus, religious involvement or importance among lower SES young, Black women may be associated with somewhat higher reproductive or contraceptive knowledge than less religious Black women with lower SES.

### 4.1 This Study’s Approach

To address our research questions, we take a concurrent mixed methods approach, simultaneously analyzing two kinds of data. In one analysis, we use survey data to estimate relationships between religiosity and reproductive and contraceptive knowledge across and within different levels of parental education and race. This is based on predictions outlined above.

However, given the somewhat limited nature of theory and prior research on how religious influence on sexual behavior, attitudes, or knowledge might vary by both social class and race, we employ a concurrent analysis of qualitative data from interviews with young women, grounding those analyses in the concept of “sexual projects” (Hirsch and Khan 2020: xiii). In their book *Sexual Citizens*, Hirsch and Khan (2020) investigate power, sex, and sexual assault on college campuses, using the concept ‘sexual project’ to help explain how college students navigate their sexual desires and experiences in college. They define a sexual project as “encompass[ing] the reasons why anyone might seek a particular sexual interaction or experience” (Hirsch and Khan 2020: xiii); a sexual project may involve not only work to have sex in a particular context (i.e. in a relationship versus hooking up) but also work done to avoid sex. This concept usefully captures reasons people have for pursuing sex (or not), what they desire to gain from sex, their desired sexual partners, and more. It also encompasses what people experience and how they make sense of their experiences. At the same time, students are trying to accomplish other projects, such as a college project, which might include studying, partying, finding a soulmate, and more (Khan et al. 2018; Hirsch and Khan 2020). In our case, we focus on relationships between ongoing sexual projects and religious projects. We compare the ways these projects are intertwined (or not) for young women with different social class and racial experiences.

Below, we describe the data, methods, and findings for each type of data. We present the quantitative data and analysis first, and then the qualitative data and analysis. We conclude by synthesizing the findings from both investigations to offer more nuanced insights into the relationship between religion and young women’s understandings of sex, reproduction, and contraception when factoring in social class and race.

## 5. Quantitative Data and Findings

### 5.1 Relationship Dynamics and Social Life (RDSL) Survey Data

The survey data we use come from the RDSL study and its baseline, in-person survey of young women (ages 18–19) from one county in Michigan in 2008–09 (Barber, Kusunoki, and Gatny 2011). Participants were randomly sampled from the Michigan driver’s license and personal identification card database and recruited to participate in an initial, face-to-face interview. The response rate for the baseline interview was 84 percent, resulting in a total of 1,003 young women. We use these data because they include unique measurement of reproductive and contraceptive knowledge combined with measures of biblical literalism, religious service attendance, and personal religiosity. Using listwise deletion for any participants missing responses to any of the questions used in our analyses results in an analytic sample of 940.

**Figure 1.**
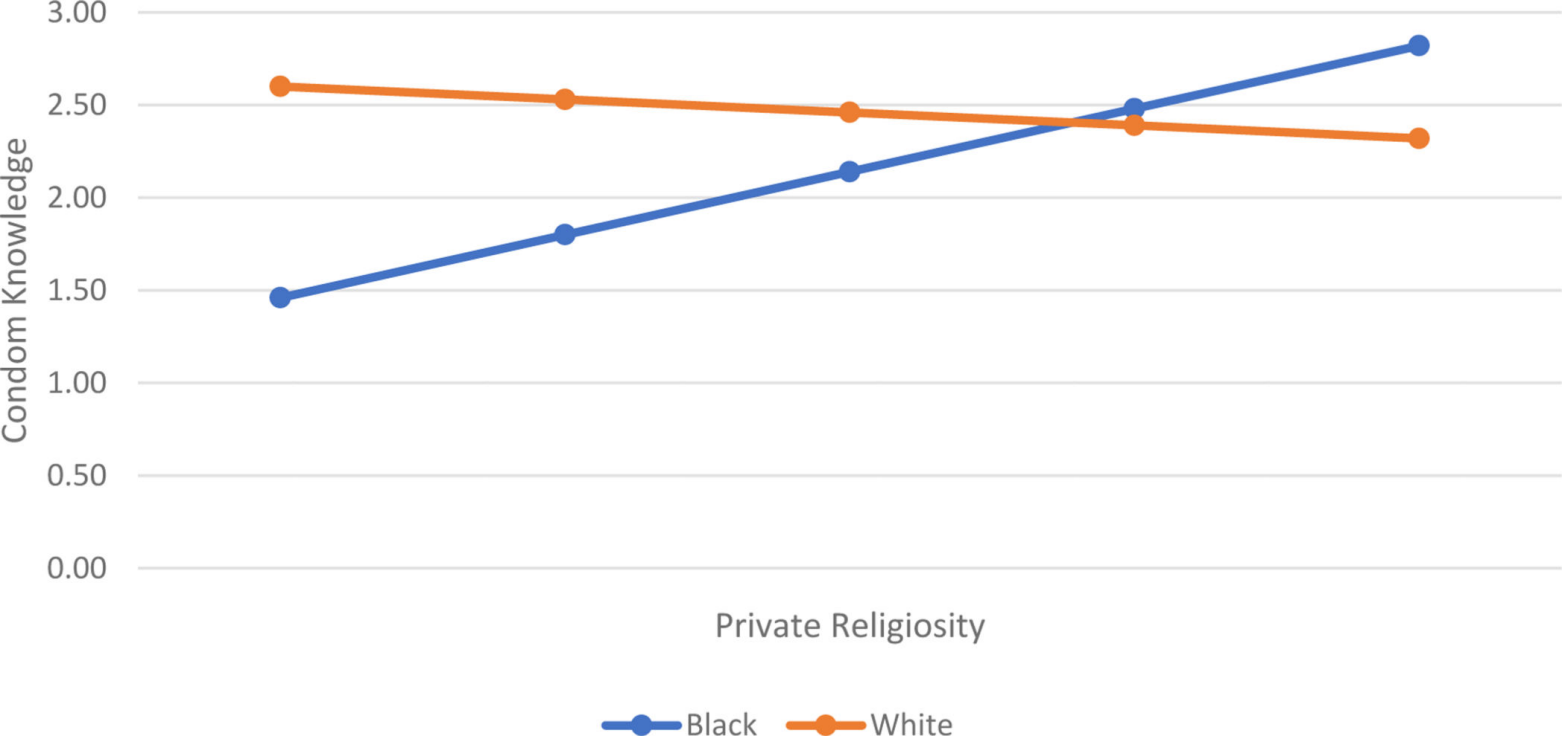
Associations between Private Religiosity and Condom Knowledge, by Race, among Higher Parental Education Group (RDSL)

Our dependent variables for the survey analysis are two indices—one measuring *female reproductive biology knowledge* and one measuring *condom knowledge*. Female reproductive biology knowledge is the sum of correct answers to the three true/false questions listed in Figure 1 (range = 0 – 3). Condom knowledge is the sum of correct answers to the three true/false questions about condom usage listed in Figure 1, but because so few participants got zero or one question correct, as compared to two or three, we merged the 0 and 1 categories together. Therefore, the range of values for condom knowledge is 1 to 3, with “1” representing those who missed at least two questions, “2” representing those who missed only one, and “3” representing all correct answers.

Our key independent variables are three measures of religiosity. First, we use a measure of *biblical literalism*. Participants were instructed to, “Please tell me if you strongly agree, agree, disagree, or strongly disagree with this statement. The Bible is God’s word, and everything happened or will happen pretty much as it says.” Responses were coded so that a higher score indicated more agreement with the statement, or higher biblical literalism. Second, we use a measure of *personal religiosity* that is the average of responses to two questions—one about the frequency of praying alone and one about how important religious faith is to the participant. This variable is coded from a low of *1* to a high of *5*. Finally, for *religious service attendance*, participants were asked, “How often do you usually attend religious services - would you say several times a week, once a week, a few times a month, once a month, less than once a month, or never?” Response were coded from *1* = never to *6* = several times a week.

Because we are focused on how the relationship between religion and reproductive or contraceptive knowledge might vary by social class, we created a dichotomous measure of parental education that divides the participants into two groups – those who do not have a parent with a four-year college degree (71 percent), and those who do have at least one parent with a four-year degree (29 percent).

Our models include three control variables: age, race, and family structure. Most participants were either 18 or 19, but nine percent were 20 by the time of the survey interview, even though they were sampled at age 19. We therefore use all three yearly age categories. We use responses to a question asking which racial/ethnic groups best describe one’s background to create a two-category race variable (0 = self-identified as White; 1 = self-identified as Black or African-American). Given our focus on race differences, all of our analyses exclude any RDSL respondents who identified with another racial or ethnic group. Family structure is measured with a set of questions that ask with whom a participant has ever lived, and then which of those people they report living with, “the majority of the time when you were growing up.” We use three categories—those who report living with two parents (biological, adoptive, or step), with one single, biological parent, or with others, for most of the time growing up. Because we are already considering multiple intersections (race, social class, and religion), we do not include sexual orientation in this study. Furthermore, we cannot provide insights into how gender may intersect with these other axes of inequality, as we have only women in our sample.

We use ordinary least squares regression to estimate the relationship between our religion measures and the reproductive and condom knowledge scores, looking for how these relationships might vary by parental education or race. To ease the interpretation of what amounts to two-way interactions in some of our analyses, we present models separately by parental education level, and then test religion by race interactions within those groups. To indicate one aspect of social class, we rely on parental education. We divide the sample into two groups, those who have at least one parent with a four-year college degree or higher and those whose parent/s have less education.

For descriptive results regarding our key variables across the two levels of parental education, see Table 1. As indicated in the far-right column, there are six variables from our analyses that vary significantly between the two groups. For example, the young women who have a parent with a four-year college degree score significantly higher on the female reproductive biology knowledge measure than those who do not have a college-educated parent. However, there is no statistically significant difference in the condom knowledge score across the parental education divide. The young women with at least one college-educated parent also attend religious services more often, are more likely to identify as White (compared to Black or African American), and are more likely to have spent most of their childhood living with two parents than those who do not have a parent with a four-year college degree.

### 5.2 Findings

First, we test whether female reproductive biology knowledge is related to religious characteristics, and how that relationship varies by parental education and/or race (see Table 2). Our lone substantive finding from this table is that having a more literalist view of the Bible is negatively related to female reproductive biology knowledge for young women who have a parent with a four-year degree, but that relationship does not hold for young women in the lower parental education group. For each unit higher a young woman’s value is on the biblical literalism measure, she has, on average, a .30 lower score on reproductive knowledge. Further, someone who strongly agrees that the Bible should be interpreted literally will miss, on average, one question more (out of the three) than someone who strongly disagrees.

For female reproductive biology knowledge, we find no interactions between religion and race. The biblical literalism finding does not seem to depend on the racial identity of the young women. Furthermore, neither private religiosity nor religious service attendance seem statistically significantly related to female reproductive biology knowledge for these women.

**Figure 2.**
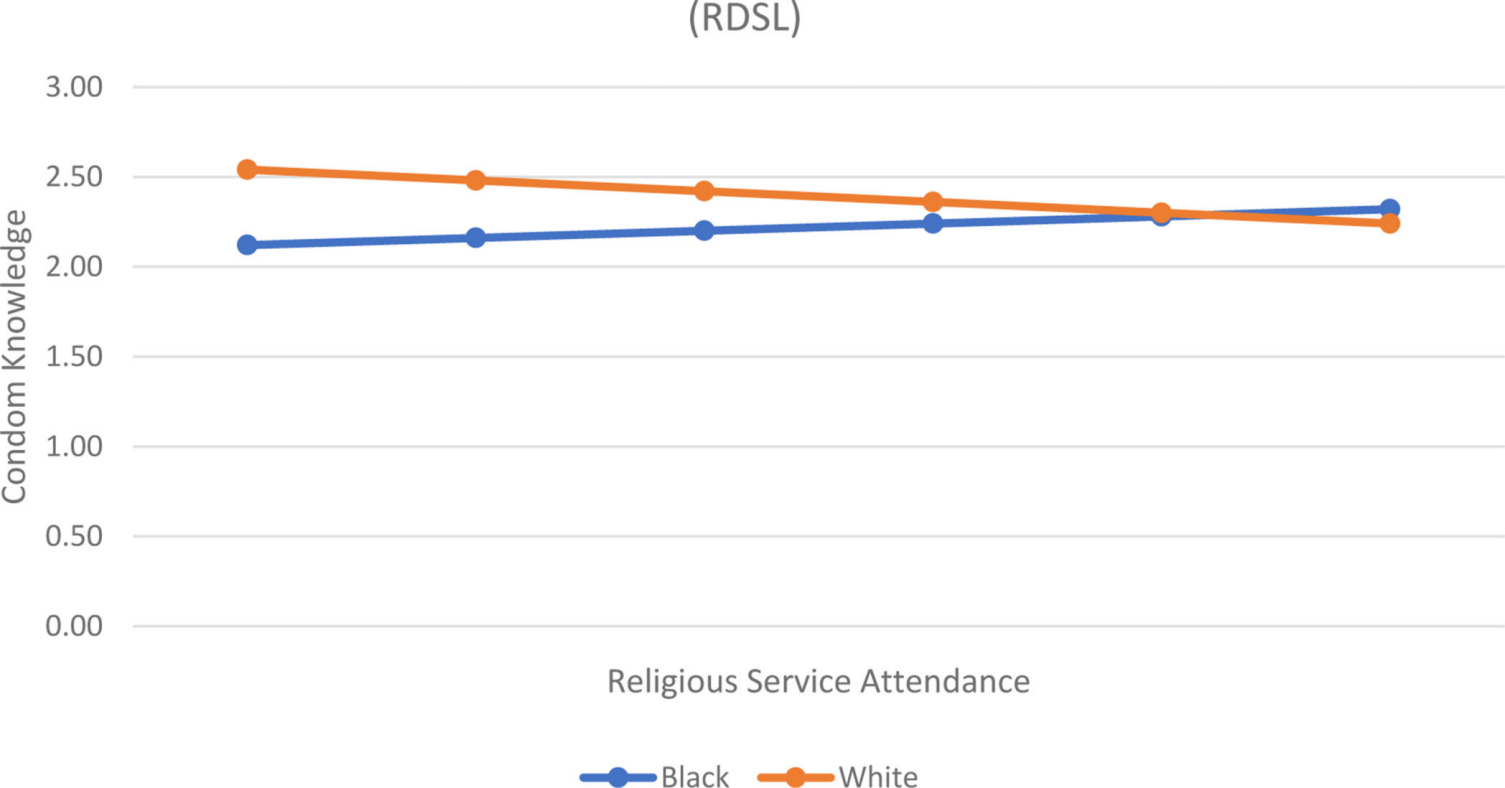
Associations between Religious Service Attendance and Condom Knowledge, by Race, among Lower Parental Education Group (RDSL)

Table 3 shows the same models predicting a different outcome – condom knowledge. With this type of knowledge, biblical literalism does not play a role. In fact, none of the religion measures are statistically significantly related to condom knowledge for either level of parental education. However, Model 2, for both groups, suggests some interesting race differentials in how religion relates to condom knowledge. First, for young women who do not have a parent with a four-year college degree, religious service attendance is positively related to condom knowledge for Black women and negatively related for White women (See Figure 1). Second, for young women with at least one parent with a four-year degree, private religiosity is positively related to condom knowledge for Black women and negatively related for White women (See Figure 2).

## 6. Qualitative Data Analysis and Findings

### 6.1 National Study of Youth and Religion (NSYR) Interview Data

The qualitative data we use to more broadly explore overlap in the sexual and religious projects of women from different social class and racial groups are the NSYR semi-structured interviews. The NSYR began with a nationally representative sample of U.S. 13–17 year-olds in 2002 (*N* = 3,290). Following the initial NSYR telephone survey, a quota sample of survey respondents (n = 267) participated in in-person, semi-structured interviews covering a range of topics. The interviews were recorded and later transcribed. The NSYR includes three additional waves of survey and semi-structured interview data (in 2005, 2008, and 2013), following these same participants’ transition to adulthood. Our analysis focuses on female interview participants who were between the ages of 18 and 20 for their Wave 2 or Wave 3 interview. This puts them at the same ages, around the same time, as the RDSL survey participants (who were ages 18–19 in 2008–2009). One key difference between the two samples is that the NSYR interview participants come from across the U.S. while the RDSL survey participants resided in one county of Michigan. However, as described below, our analysis of the qualitative data focuses on comparing young women across groups sorted by religiosity, parental education, and race. We posit that although the one county in Michigan differs demographically from the entire nation, the experiences of young women within each subgroup is likely similar enough in other areas of the U.S. to make this analysis informative. We reflect further on potential limitations of the non-nested samples in the conclusion.

The interviews we analyzed come from 44 Black and White women from the NSYR, between the ages of 18 and 20, who identified as Mainline Protestant, evangelical Protestant, or Catholic at the time of their interviews. We closely read the entire interview transcripts to examine how these young women describe and enact their religious and sexual projects. By considering the entire interview, and not just the sections where interviewees are asked directly about their thoughts on sex, contraceptive use, pregnancy, and STIs, we can see how their religious and sexual projects emerge when discussing all aspects of their lives. We wrote detailed memos for each woman in which we described her social class, family experiences, religiosity, sexual behaviors and attitudes, and thoughts on contraceptive use, pregnancy, and STIs.

We then grouped women based on their race, parental education, and religious backgrounds which resulted in six groups. Due to having few high SES Black women in the subgroup on which we focus our analysis, we do not examine religious and sexual projects at that particular intersection of identities. After rereading each subgroup closely, we identified the central sexual and religious projects that emerged for that particular set of women, which we summarize in Table 5. We did so by identifying and comparing patterns that emerged in four main areas: how they talked about contraceptive and reproductive knowledge, including common sources of information; how they describe their religious beliefs and practices; their perceptions of the relationship between religious teachings and their own beliefs/behaviors; and their thoughts on contraceptive use and sexual behaviors among their peers.

Important differences emerged between social classes, race, and levels of religiosity. For example, we observed that regardless of religiosity, high SES white women were similar in their sources for contraceptive knowledge (school and doctors) and in their unwillingness to impose their own values on others; in contrast, low SES white women rely more on knowledge gained from their peers or from becoming sexually active themselves. When we then look at religious differences within these groups, we see that religion acts differently, with highly religious, low SES women asserting that all teenagers should practice abstinence and highly religious, high SES women emphasizing abstinence is right for them but may not be embraced by everyone.

To facilitate presentation of the full complexity in these young women’s expressions of their sexual and religious projects, we constructed composite narratives, which allow us to describe one composite person to represent each group. Recently, scholars have used composite narratives as an additional step to protect participants’ confidentiality and identity, particularly when studying public figures (Willis 2017) or vulnerable interviewees who could face serious harms should they be identified (Elizabeth 2017; Piper and Sikes 2010); however, this technique also facilitates more complete and holistic comparisons across groups, especially when the nature of the interview (in this case a highly structured, but open-ended interview) results in shorter responses per question, but greater range across domains.

Willis (2019) identifies several key practices for methodically constructing composite narratives, which we follow here to ensure rigor, precision, and integrity in this form of data presentation. First, we ground each narrative in patterns observed across 3–7 interviews. Second, we include actual quotations from the interviews, so that readers can see firsthand the language used by participants. Third, we clearly distinguish between interviewees’ interpretations of their lives and our own analysis. Although a relatively new way to present qualitative findings, we believe that this approach benefits us in several ways. For one, we can capture the complexity of these groups of women without relying on an ideal case, and we can thus avoid the rigidity of some typologies (Malvicini 1999; Willis 2019). For another, we can more thoughtfully go beyond details about their contraceptive knowledge and religiosity to include other aspects of their lives, which strengthens our analysis of their religious and sexual projects without obscuring the key comparisons (Elizabeth 2017; Upton-Davis 2015).

### 6.2 Findings

Our analysis of the broader linkages between religious and sexual projects and how those vary depending on parental education level and race reveals three interesting themes. First, regardless of social class or race, young women who are religious openly state an ideal of premarital abstinence. What differs is how attainable they view that ideal for themselves and the extent to which they are prepared to prevent pregnancy or STIs. We start by showing the role of religion for white women with college educated parents and then compare them to white women whose parents did not graduate from college. This reveals how connections between religious projects and sexual projects operate differently by social class. We then compare these experiences to Black women with parents who do not have a college degree, examining how religion plays a unique role in these women’s lives compared to the previously discussed white women with parents who have no college degree. Through these comparisons we illuminate racial variation in how religion operates in lower SES contexts.

#### 6.2.1 White Women with Higher Parental Education: Religion Alters Personal Strategies

Rachel^1^, a young white woman, is currently in her first year at college, where her parents provide substantial financial help; after college, she anticipates either pursuing a professional degree or immediately beginning her career. Her parents have been married since before she was born, both have college degrees, and she gets along well with them. They participate in religious activities as a family, such as attending church and having occasional conversations about faith. Rachel reports having drawn on specific religious teachings to make decisions about what to do, and she explains that she tries to apply biblical lessons to her own life. She generally believes that her religion is the right one and appears uncomfortable wrestling with ideas of moral relativism, but she is hesitant to insist her ideas of right and wrong are universally true:

I think, like, if you have like faith in God, in Jesus and like he rose from the dead, then I definitely think that you’re going to have morals different than like a person that’s secular, and I don’t really… [pause] I know there’s so many beliefs out there, I don’t really, I don’t know, just like to other people what’s wrong is definitely not going to be wrong. But to me, like it would be wrong. I don’t know how to describe it.

Ultimately, her religious project is grounded in having the right beliefs and doing her best to embody those beliefs in her daily practices, although she believes there is room for improvement.

Accordingly, Rachel plans to wait until marriage to have sex, as this is what she believes her religion teaches. However, she is not particularly interested in imposing her values on others, explaining, “You can’t force people to abstain from sex.” She is a little concerned about the rise of casual sex, and in particular, the lack of contraceptive use: “I feel like people are being so stupid about [sex] … like not using a condom, not using any form of birth control even.” She sees the value in sex education, and explains that most of her contraceptive and reproductive knowledge comes from schools and from doctors. Additionally, she believes her family and her church support her decision to practice abstinence, and she does not indicate having any plans of her own for contraceptive use. Rachel’s sexual project is connected to her religious project; she practices abstinence as a way to accomplish her religious project, which teaches abstinence until marriage, by aligning her beliefs with her behaviors. However, she is not oblivious to the patterns of sexual activity around her, and she believes that others who choose to become sexually active should take steps to avoid pregnancy. Finally, although she feels positively about contraception for others, she may lack practical knowledge about both female reproductive and condom knowledge, given she does not currently perceive a need for that knowledge in her daily life.

Like Rachel, Emma is a white woman in her first year of college. She grew up in a suburban neighborhood, and frequently vacationed with her married, college-educated parents. She is somewhat financially literate, evaluating potential college majors based on how quickly she could pay off any debt. Growing up, she had some religious exposure, attending occasionally with her parents. Now, her religious project can be characterized as nominal: she is happy to attend services for major holidays, and she identifies as Christian, but she explains that religion does not shape her daily life or her decisions.

Emma first became sexually active with her high school boyfriend; she explains, “You are ready to have sex when you are mature and can have safe sex.” Indeed, this emphasis on maturity and responsibility surfaces frequently in the interview section on relationships, sex, and contraception, revealing a sexual project emphasizing choice in becoming sexually active and agency in pregnancy and STD avoidance. For example, when she noticed that she had become inconsistent in her condom use, she sought out birth control pills instead. Although both Emma and Rachel have consistent access to doctors for reproductive healthcare, Emma sees her doctor as a helpful resource for knowledge that is important to her sexual project, whereas Rachel relies more on her parents’ normative ideas about sex, which are informed by their shared religion, to inform her sexual project. In contrast, Emma emphatically states that her ideas and knowledge around sex and pregnancy “did not come from my parents, that’s for sure;” she views them as generally more conservative than she is, so she relies more on friends, her own research, and her doctor for information.

Rachel and Emma are similar in their support for those who choose to be sexually active being knowledgeable and prepared to prevent pregnancy; they also enjoy similar access to sex education via schools and the doctor’s office. However, they differ in their own personal sexual projects: Emma is sexually active while Rachel is not. Their discussions of sex education classes in school, their access to the Internet for research, and their ability to see a gynecologist all speak to their shared high socioeconomic status and could easily translate to higher levels of reproductive knowledge than their lower SES peers. However, this SES advantage is somewhat eroded for women who are more religious, particularly those who may be Biblical literalists. Although Rachel does not directly invoke her religious beliefs when sharing her thoughts on others’ contraceptive use, she is very clear that she agrees with her religion’s teaching on sex (abstinence until marriage), and since she is practicing abstinence herself, she may see no need to seek information about reproduction or contraception. This analysis suggests that her religious project, which reinforces her sexual project of abstinence, may be subtly and/or inadvertently discouraging her from seeking additional information.

#### 6.2.2 White Women with Lower Parental Education: Religion is Neutral When Misinformation is High and Opportunity Costs are Low

Lauren, a white woman living in an under-resourced area, recently graduated from high school. She now works a part time job and aspires to attend community college, although she is unsure when she will be able to afford classes. She attends church regularly, usually with her mother (who is a single parent), and she tries to engage in some private practices, such as prayer. Her religious project emphasizes individual morality and doing what is right in the eyes of God; she mentions the Bible as a source of authority, and occasionally cites biblical teachings when talking about her decision-making processes. As a result, her sexual project is closely intertwined with her religion project, as Lauren believes that to be a good Christian is to abstain from any sexual activity before marriage.

Because she understands sex in moral terms, Lauren believes that ideally everyone should wait until marriage to become sexually active; the strong link between her religious and sexual projects means that in her mind, abstinence is not just the best choice for her: it is the best choice for everyone. She explains, “I think they have to wait ‘til they are married … I’d say kissing [is okay]. And then, anything that’s gonna leave you to wanting to have sex is wrong.” Furthermore, her sexual project is reinforced by religious teachings about procreation and the centrality of sex to reproduction; as a result, she believes that ultimately “you are supposed to want to reproduce and have kids,” and she is somewhat skeptical of the efficacy of birth control. She explains:

I’m actually on birth control right now, but, you know, it’s just to try and get started on it. I don’t really, I mean I see the need in it, but then I don’t see the need in it I guess. I feel like if you’re gonna get pregnant, you’re gonna get pregnant.

Valuing procreation may not necessarily translate to reproductive knowledge; in fact, because she closely connects sexual activity to reproduction, she may not feel motivated to learn more about pregnancy or contraceptives, given pregnancy is perceived as somewhat unavoidable, even if on birth control pills. Thus, pregnancy is not necessarily something she will seek to avoid once sexually active. Furthermore, unlike the higher SES white women, Lauren cites neither school nor doctors as sources of contraceptive knowledge; instead, she points to her own and her friends’ experiences as providing useful insights into different types of birth control and STD risks.

Melanie, a young white woman, recently graduated from high school, where she averaged Bs and Cs. Due to her parents’ divorce, she has experienced some instability in her housing, and she is currently living with her mom and her mother’s long-term boyfriend. She works part-time at a fast food restaurant as she figures out her next steps. Although she attended church intermittently with her family as a child, she does not attend now, and she does not believe that religion impacts her daily life. When asked about her beliefs, she provides short, generic answers that suggest a lack of reflection. Ultimately, she concludes after that section of the interview: “I guess I’m not very religious.” At the same time, however, she does not reject Christianity, so her religion project could be characterized as Christian in name only; she provides a religious affiliation at each wave of the survey, but consistently says religion is “not very important.”

Melanie became sexually active at 16 with her then-boyfriend. She says of sex, “I think if [teenagers] are comfortable with themselves then they should do what they want. You know, if they feel like they should have sex, then by all means go ahead and do it.” However, she believes it is better to be in a relationship first, which is the only context in which she has had sex. In terms of contraception, although she thinks it is good and people should use it, particularly if they are having sex outside of a committed relationship, her own use is inconsistent, which led to a recent pregnancy scare. This scare initially prompted her to use contraception more consistently, but at the same time, she expresses ambivalence toward pregnancy, explaining: “we were going to try and use the condom as much as possible, because we both know that we’re not mature enough to have a child. We’re not, um, financially ready to have a child.” They understand there is a chance of pregnancy, and that, “it may happen and when the time comes, and if it does happen, we will handle it responsibly and maturely.” She explains that her knowledge comes from being sexually active, which echoes Lauren’s observations. Thus, it may be that social class impedes access to contraceptive knowledge if under-resourced schools provide inadequate sex education or if young adults lack access to healthcare.

Although pregnancy is a primary concern, she also worries about STDs, which reinforces her commitment to having sex only when in a relationship. She explains: “You never know what you might catch from somebody, you never know who they’ve been with and I’m a strong believer that if you sleep with one person it’s just like sleeping with every person they’ve already been with.” Overall, her sexual project emphasizes choice (for herself and for others) in becoming sexually active and prioritizing relationships as the context for having sex. The relationship is implicitly viewed as minimizing the consequences of unprotected sex, as she sees a monogamous male partner as less likely to have an STD and as able to communicate in the event of an unintended pregnancy; given this, consistent contraceptive use is not central to this particular sexual project.

#### 6.2.3 Black Women with Low Parental Education: Religion as a Resource for Social Mobility

Monique is a 19-year-old Black woman who recently graduated from high school. She currently lives with her mother, with whom she reports a fairly good relationship, and she regularly visits her grandparents, who live across town. She works a part time job as she thinks about her future; she hopes to attend college or to open her own business, but she does not have a concrete plan for achieving either goal. Monique attends church regularly with her extended family, saying “all my family goes to church” at a historically Black congregation. She enjoys attending and talking with some of the adult members. Furthermore, she believes that religion shapes her everyday life. Her religious project involves maintaining a personal relationship with God, attending church, and “calling on the Lord” as she goes about each day. Although she reports a high level of religious salience, she does not generally talk in terms of religious or moral absolutes.

When talking about sex, Monique notes that “like 99% of my friends are active,” and she recently became sexually active because she felt she “was ready to accept any consequences that I might have come with my actions.” However, she does not anticipate having consequences, because she will use protection to avoid an unintended pregnancy, which she views as a major issue for young women and as something that cannot be hidden from others (unlike an STD). These ideas reflect a sexual project that emphasizes maturity, responsibility, and safe sex. Recognizing that there are risks to having sex, and then taking steps to mitigate those risks, reflects maturity and suggests that one is ready to become sexually active. Finally, she believes that abstinence until marriage is impractical advice that is disconnected from contemporary realities, suggesting awareness that abstinence is a common religious teaching but that she does not directly encounter it. She also cites a range of sources for her contraceptive knowledge, including her own research on the Internet and conversations with trusted adults. Perhaps because she encounters a range of information, Monique does not personally experience conflict between her religion and her sexual projects. She is, however, somewhat vague in her discussions of contraceptive use, suggesting that at times her behaviors at times contradict her commitment to maturity and responsibility.

A 19-year old Black woman, Naomi has an 18-month old son; they both live with her mother and several extended family members in a single-family home. She recently earned her GED, and she is working part-time while relying on family members to care for her son. She is no longer with her son’s father, but he lives in the area and tries to stay involved in his son’s life. She talks about potentially taking classes at a local community college, but cost is a major barrier. Naomi holds basic religious beliefs, such as belief in God and Jesus, but her thoughts on morality and decision-making are not grounded in a religious framework. In terms of religious participation, she says, “I barely go to church.” However, she is quick to explain that she does not doubt God’s existence, suggesting a religious project of nominal Christian; maintaining the core “correct” beliefs is important, but she does not use religion as a resource in her life.

Unlike Monique and any of the other young women in the previously described groups, Naomi is actively worried about STDs, particularly HIV/AIDS, more so than about unintended pregnancies. Answering a question about how much pregnancy and STDs are concerns for teenagers, she says:

It’s 100% [a] concern because disease is spreading fast and you can ask a person if they have something and they can lie to you and say they don’t and they could have it all the while and you make the choice to do something like that and you expose yourself in to catching it. So it’s a concern to every, it’s 100% concern.

Although she repeatedly details this concern and claims that some people actively try to spread their STDs, she is less knowledgeable on how to safely protect herself, often portraying sex as inherently risky, even if one were to use condoms or birth control. She does not cite formal sources of knowledge (such as a doctor or a class), but instead references her own experiences and conversations with friends. She personally prefers condoms, although she uses them inconsistently, and she says, “I don’t really know about birth control, but I heard about it.” She expresses skepticism about the efficacy of all methods for preventing pregnancy, which contributes to her general ambivalence towards a second pregnancy. Accordingly, her sexual project views sex as a natural act that is inherently risky; combined with her skepticism around birth control and the absence of any sex education, her perspective that sex always carries risk likely contributes to her inconsistent contraceptive use and may make it less likely that she will seek additional information.

Comparing Monique and Naomi’s lives to the white women whose parents also have no college education reveals how religious projects and their links to sexual projects vary by race, at this level of socioeconomic status. Monique, who is more religious, articulates a desire to avoid pregnancy, and she is confident in her ability to do so, suggesting a certain level of knowledge. Furthermore, at church she may encounter more emphasis on overall health and well-being than on premarital abstinence, which may somewhat encourage her to know more about reproduction and contraception. In contrast, Naomi is more ambivalent toward pregnancy, and although she has had a child and is actively worried about STDs, her condom use remains inconsistent.

## 7. Conclusions

In this paper, we have used a concurrent mixed methods approach to analyze how religion plays a role in young women’s contraceptive and reproductive knowledge and how that role varies by race and social class. Using survey data from the Relationship Dynamics and Social Life study and interviews from the National Study of Youth and Religion, we use a complex religion framework in which we consider race and class simultaneously to more precisely specify the impact of religion on these two types of knowledge. Building on these findings, our study offers three major contributions.

First, our study contributes to the existing literature showing that operationalization of religion matters for sex-related outcomes, as we find not only that distinct measures of religion were associated with reproductive and contraceptive knowledge differently, but that the associations further varied by race and class. For example, we found that biblical literalism was only associated negatively with reproductive knowledge for women whose parents had a college degree (regardless of race), and yet biblical literalism was not related to level of contraceptive knowledge for any of the groups we examined. Instead, what seems related to contraceptive knowledge were the two other religious measures – but which one was significant varied by level of parents’ education and the direction of the association varied by race. Among women whose parents had more education, attending religious services more frequently was associated with greater condom knowledge for Black women but less knowledge for White women, whereas among women whose parents did not have a college education, private religiosity predicted more knowledge for Black women but less for White women.

Thus, our findings reaffirm the importance of using multiple measures of religion and distinct scales for reproductive and contraceptive knowledge. Reproductive knowledge may connect more closely to Biblical literalism given Christian messages about procreation, while condom knowledge may align more closely with personal religiosity and religious service attendance, as all three may result more from individuals’ agency and efforts. Furthermore, at first glance, although social class may shape what measure of religiosity is most salient, without also examining race, we would mistakenly assume that religion similarly impacted all women who shared the same social class background. This nuance in the results speaks to the importance of a complex religion approach.

Accordingly, our second significant contribution is to the burgeoning complex religion literature. Situating our work in the complex religion framework required us to simultaneously consider overlapping identities. By considering the intersection of race and class among young women, we can more accurately specify the relationships between different dimensions of religion and both contraceptive and reproductive knowledge. Religion is not universally protective for women when it comes to this type of knowledge, and at times religious involvement is related to having less knowledge, so better understanding how religion plays a role could help with interventions to minimize unintended pregnancies and to ensure women have the knowledge they need to engage in safer sex practices. To further develop the complex religion framework, future studies should consider a comparative historical approach that would facilitate a closer examination of how race has intersected with religion and contraceptive knowledge/use over time. Building on our research, two clear directions emerge: scholars might consider, first, how historical differences in Black and White religious organizations contribute to different approaches to sexual health and reproductive justice today, and second, how the history of discrimination and racism in medical communities (particularly when it comes to Black women’s reproductive health) intersects with Black women’s contemporary experiences of racism and their religious projects to inform their access to reproductive knowledge and healthcare (Prather et al. 2018; Rosenthal and Lobel 2018; Tekeste et al. 2018).

Finally, taken together, our quantitative and qualitative analyses suggest that looking more holistically at young women’s lives will better reveal how they accomplish their religion and sexual projects. Our research reveals the need for future studies examining the relationship between religious and sexual projects; for example, we found that for White women with less educated parents, their sexual project of abstinence allowed them to accomplish their religious project of being what they consider a good Christian. Rather than asking only targeted questions about religion and sexual knowledge, researchers may want to investigate more broadly: to what extent do people experience their religious and sexual projects to be in conflict? How do people navigate and make sense of potentially conflicting messages from their religion, their parents, their peers, and more? Future studies would do well to investigate reproductive and contraceptive knowledge by inquiring more specifically about religious teachings on sex and contraception, and then contextualizing those messages in women’s broader experiences and identities, including but not limited to race, social class, sexual orientation, friend groups, cultural beliefs about sex, and more.

## Figures and Tables

**Table 1.** Descriptive Statistics by Parent Education Level (RDSL)

		Lower Parental Education (n=669)		Higher Parental Education (n=271)		

*Variables*	*Range*	*Mean*	*SD*	*Mean*	*SD*	*Difference*
Female reproductive biology knowledge	0–3	1.65	0.90	1.87	0.96	*
Condom knowledge	1–3	2.35	0.68	2.40	0.71	
Biblical literalism	0–1	0.24		0.24		
Personal religiosity	1–5	3.28	1.21	3.30	1.19	
Religious service attendance	1–6	2.97	1.64	3.48	1.67	*
Age						
18 years	0–1	0.40		0.44		
19 years	0–1	0.51		0.47		
20 years	0–1	0.09		0.09		
Race						
Black (ref = White)	0–1	0.41		0.21		*
Family Structure						
Two parent family	0–1	0.45		0.71		*
Single biological parent only	0–1	0.45		0.25		*
Other	0–1	0.10		0.04		*

Note:

* p < .05

**Table 2.** True/False Questions Comprising the Survey Outcomes (RDSL; *n* = 960)

Reproductive Female Biology Knowledge	Percent Answering Correct
• The most likely time for a woman to get pregnant is right before her period starts (*false*)	39%
• In general, a woman is most likely to get pregnant if she has sex during her period, as compared with other times of the month (*false*)	64%
• When a woman misses more than two days of birth control pills, she should use another birth control method (*true*)	68%
*Condom Knowledge*	
• Even if the man pulls out before he ejaculates, even if ejaculation occurs outside of the woman’s body, it is still possible for the woman to become pregnant (*true*)	81%
• When putting on a condom, it is important to have it fit tightly, leaving no space at the tip (*false*)	61%
• As long as the condom fits over the tip of the penis, it doesn’t matter how far down it is unrolled (*false*)	93%

Note: Correct answers in parentheses

**Table 3.** OLS Regression Models of Relationships between Religion Measures and Reproductive Knowledge Score, Separate by Parent Education, and Interactions with Race (RDSL)

	Reproductive Knowledge Score			

	*Model 1*		*Model 2*	

	Lower Parental Education (n=669)	Higher Parental Education (n=271)	Lower Parental Education (n=669)	Higher Parental Education (n=271)
*Religion Measures*				
Biblical literalism	−0.06	−0.30*	−0.23	−0.19
	(.09)	(.14)	(.15)	(.17)
Private religiosity	0.01	−0.02	0.02	−0.05
	(.04)	(.06)	(.05)	(.07)
Religious service attendance	−0.01	−0.01	−0.02	−0.02
	(.03)	(.04)	(.04)	(.05)
*Religion and Race Interactions*				
Biblical literalism * Black			0.28	−0.40
			(.19)	(.31)
Private religiosity * Black			0.01	0.18
			(.09)	(.15)
Religious serv attend * Black			0.03	0.05
			(.06)	(.10)
*Control Variables*				
Age (Ref: 18 years)				
19 years	0.14+	−022+	0.14+	−0.22*
	(.08)	(.11)	(.08)	(.11)
20 years	0.04	0.07	0.04	0.09
	(.13)	(.19)	(.13)	(.20)
Race (Ref: White)				
Black	−0.40***	−0.41**	−0.60*	−1.17
	(.09)	(.15)	(.29)	(.63)
Family Structure (Ref: Two parents)				
Single bio parent only	−0.27***	−0.02	−0.27***	−0.02
	(.08)	(.13)	(.08)	(.13)
Other	−0.31*	0.26	−0.32*	0.31
	(.13)	(.28)	(.13)	(.28)
Intercept	1.91***	2.23***	1.93***	2.31***
	(.12)	(.18)	(.14)	(.19)
R-squared	.09	.10	.10	.11

Notes: Standard errors in parentheses

* p < .05

** p < .01

*** p < .001

**Table 4.** OLS Regression Models of Relationships between Religion Measures and Condom Knowledge, Separate by Parent Education, and Interactions with Race (RDSL)

	Condom Knowledge			

	*Model 1*		*Model 2*	

	Lower Parental Education (n=669)	Higher Parental Education (n=271)	Lower Parental Education (n=669)	Higher Parental Education (n=271)

*Religion Measures*				
Biblical literalism	−0.10	−0.05	−0.06	−0.02
	0.07	0.11	0.12	0.13
Private religiosity	−0.003	−0.0001	−0.01	−0.07
	0.03	0.05	0.04	0.05
Religious service attendance	−0.01	−0.03	−0.06+	−0.02
	0.02	0.03	0.03	0.04
*Religion and Race Interactions*				
Biblical literalism * Black			−0.05	−0.25
			0.15	0.24
Private religiosity * Black			0.06	0.44***
			0.07	0.11
Religious serv attend * Black			0.09*	0.06
			0.04	0.08
*Control Variables*				
Age (Ref: 18 years)				
19 years	−0.04	−0.03	−0.04	−0.03
	0.06	0.09	0.06	0.08
20 years	−0.11	0.13	−0.11	0.17
	0.10	0.15	0.10	0.15
Race (Ref: White)				
Black	−0.19**	0.03	−0.69**	−1.81
	0.07	0.12	0.22	0.48
Family Structure (Ref: Two parents)				
Single bio parent only	−0.02	−0.14	−0.03	−0.15
	0.06	0.11	0.06	0.10
Other	0.05	0.02	0.02	0.10
	0.10	0.22	0.10	0.22
Intercept	2.53***	2.55***	2.66***	2.72***
	0.09	0.14	0.11	0.15
R-squared	.03	.02	.05	.08

Notes: Standard errors in parentheses

* p < .05

** p < .01

*** p < .001

**Table 5.** Sexual Projects across Race, Parental Education, and Religiosity

		Religious	Not religious
Black women	Higher parental education	**No black women in this category of parental education are very religious, so we do not analyze this group.	Sexual activity is connected to maturity and to adulthood project - must be able to understand risks (pregnancy and STDs) and to take them seriously.
	Lower parental education	Sexual activity is connected to maturity and to adulthood project, rather than to religious project (which is still salient).	Sexual activity is framed as risky, yet contraceptive use is inconsistent.
White women	Higher parental education	Abstinence for them to accomplish their own religious project, but choice and pregnancy avoidance for others.	Emphasis on choice (for themselves and others) in becoming sexually active and pregnancy avoidance
	Lower parental education	Abstinence is understood in moral/absolute terms as part of their religious project, and birth control is viewed with skepticism.	Emphasis on choice (for themselves and others) in becoming sexually active and protection against STDs and pregnancy.

## Footnotes

1 As a reminder, we are presenting composite cases, comprised of the experiences and expressions of multiple women in each category, and as such, the names are pseudonyms.
